# Dietary diversity, undernutrition and associated factors among pregnant women in Gindeberet district, Oromia, Ethiopia: a cross-sectional study

**DOI:** 10.1186/s40795-023-00773-2

**Published:** 2023-10-13

**Authors:** Segni Mulugeta Tafasa, Jiregna Darega, Nagasa Dida, Feyisa Dudema Gemechu

**Affiliations:** 1 Department of Public Health, Institute of Health Sciences, Wallaga University, Nekemte, Ethiopia; 2 Department of Public Health, College of Medicine & Health Sciences, Ambo University, Ambo, Ethiopia

**Keywords:** Dietary diversity, Undernutrition, Pregnant women, Gindeberet district

## Abstract

**Background:**

Appropriate levels of dietary diversity are essential for proper physiology of human being and it is crucial to consume healthy foods at every phase of life, especially during pregnancy. Inadequate dietary diversity and malnutrition are risk factors for low birth weight, intrauterine growth reduction and small for gestational age. This study was aimed to assess dietary diversity, undernutrition and associated factors among pregnant women in Gindeberet district, Oromia, Ethiopia, 2020.

**Method:**

A community based cross-sectional study was conducted among 627 pregnant women in Gindeberet district selected by systematic random sampling from October 10/2020– November 10/2020. Data were collected through interviewer administered questionnaires. The collected data were coded and entered to Epi-info version 7.2.2.6 and analyzed by SPSS version 23. Logistic regression analysis was carried out to identify factor associated with undernutrition and dietary diversity. Level of statistical significance was declared at p-value < 0.05.

**Results:**

overall prevalence of inadequate dietary diversity and undernutrition were 276 (44.4%) and 110 (17.7%) respectively. Pregnant women who did not receive antenatal care (AOR = 2.32, [95% CI: 1.38, 3.90]), family size ≥ 5 (AOR: 2.93; [95%CI: 1.10, 7.79]), unprotected sources of water (AOR: 4.14; [95% CI: 1.63, 10.52]) were significantly associated with undernutrition. Rural residence (AOR = 2.59, [95% CI: 1.66–4.04]), pregnant women who did not received ANC (AOR = 2.52, [95% CI: 1.58–4.03]) and nutrition information (AOR = 1.43; [95% CI: 1.10, 2.10]) were significantly associated with dietary diversity among pregnant women.

**Conclusion:**

undernutrition and inadequate dietary diversity among pregnant women were high in study area. Source of drinking water, ANC visit and family size were significantly associated with pregnant women undernutrition. Place of residence, ANC visit and nutrition information were significantly associated with inadequate dietary diversity. Therefore, pregnant women, government, non-governmental organization and stakeholders should focus on importance of ANC visit, clean source of drinking water and adequate dietary diversity to improve nutritional status of pregnant women.

## Introduction

Undernutrition is an outcome of insufficient quantity and quality of food and frequent episodes of infectious disease or consumption of inadequate energy, protein and micronutrients to meet basic requirements for body maintenance, growth, and development [1]. Dietary diversity is the consumption of a variety of food groups over a reference period which has been accepted as an aspect of dietary quality and can show nutritional adequacy [2].

Appropriate levels of nutrients are essential for proper physiology of human being and it is crucial to consume healthy foods at every phase of life, beginning in the womb. Good nutrition is vital for any pregnancy and not only helps for health of mother, but also influences the development of the fetus and ensures that the baby grow well in infancy and beyond [3, 4].

Nutrition is a vital part of human life and its need differs with age, gender and physiological changes such as pregnancy (changes in body composition, weight gain, changes in blood composition, metabolic changes and adaptive responses) [4]. Over all energy needs increased by 13% during pregnancy. Specifically, protein need increased by 54% and vitamin and mineral need by 0-50%. To meet these high demands most nutrients needs are increased during pregnancy [5]. During the prenatal period, the fetus obtains all of its nutrients through the placenta and maternal tissues such as breast and uterus need improved energy requirements for tissue synthesis. So dietary consumption has to meet needs of mother as well as the products of conception and enable the mother to lay down stores of nutrients required for the development of the fetus [6]. Pregnant women need one extra meal every day in order to maintain good health and strength to maintain their health and the health of the baby. So, pregnant women should choose a high quality, diverse diet, consume fresh foods and prepare nutrient rich meals [3].

Women with good nutritional status during pregnancy are better able to cope with the stress of pregnancy and have good pregnancy outcome. Nutritional intervention during pregnancy would prevent poor maternal weight gain and decrease the incidence of low birth weight and preterm birth by using locally accessible and affordable diets [7]. Balanced energy and protein intake during pregnancy improves fetal growth and can minimize the risk of stillbirth and small-for-gestational-age infants [8]. Well-nourished mothers have healthier babies and a lower risk of maternal mortality and morbidity [9]. A woman who was able to consume the recommended amounts of nutrients during pregnancy will have stored enough fat to be used for herself and the fetus [10].

Maternal undernutrition is a worldwide public health problems affecting higher proportion of women in developing countries [11]. It remains as persistent and destructive health problem in low and middle-income countries. The global undernutrition among women in reproductive age is significantly higher in Africa, particularly in sub-Saharan Africa, South central and Southeastern Asia due to chronic energy and/or micronutrient deficiencies especially during pregnancy [12].

According to a study done on the burden and determinants of malnutrition among pregnant women in Africa 23.5% of pregnant mothers are living with the undernutrition problem and they might have been suffering from pregnancy complications and adverse birth outcomes related to their nutritional problems. Many women in Africa suffer from chronic energy deficiency, inadequate weight gain during pregnancy, and low micronutrient status [13]. Undernutrition is one of the most serious health problems affecting both mothers and their children in Ethiopia [14]. As evidenced by 2016, EDHS, malnutrition among women is high with 22% of them is undernourished or thin, which is linked to a maternal mortality rate of 420/100,00live birthshs [15]. Similarly different study conducted in Ethiopia showed prevalence of undernutrition ranges from 14.4 to 44.9% [16–22].

Inadequate dietary diversity is also worldwide problems. The study done in Laikipia; Kenya found 39.2% of pregnant women had low dietary diversity score [23]. Another study conducted in Nepal showed that 64.2% of pregnant women had low dietary diversity score [24]. In Ethiopia inadequate dietary diversity among pregnant women is high. Different studies conducted across the country reported inadequate dietary diversity among pregnant women which ranges from 38.8 to 69.6% [2, 17, 25–27].

Maternal nutritional status during pregnancy has significant consequences for health of both pregnant women and newborn babies. Improper nutrition intake is risk factor for low birth weight (LBW), intra uterine growth restriction (IUGR), and small for gestational age (SGA), preterm birth, stillbirths, miscarriages, growth failure, increased risk of maternal and neonatal mortality, impaired cognitive development, sub-optimal productivity in adults and reduced economic growth and other adverse outcomes during pregnancy and births [28].

Undernutrition and inadequate dietary diversity among pregnant women are commonly associated with increased demands during pregnancy and lactation, infections, socio-demographic related factors, obstetric related factors, dietary consumption related factors and environmental related factors [13, 16, 18, 20, 22, 25, 29–33].

Due to the intergenerational effect of malnutrition government of Ethiopia have been proposed initiatives such as National Nutrition Strategy (NNS) and National Nutrition Program (NNP) to tackle this problem [34] Despite this magnitude of undernutrition and inadequate dietary diversity is remained high among pregnant women. Even though nutritional status of pregnant women is important in breaking intergenerational effect of undernutrition, little is known specifically in our study area. Therefore, this study was aimed to assess undernutrition, dietary diversity and associated factors in Gindeberet district, Oromia, Ethiopia.

## Methods

### Study setting and period

The study was conducted in Gindeberet district. Gindeberet district is found at 192 km far from Addis Ababa to the west and bounded by Horo Guduru Wollega Zone to the west, by Chobi district to the south, by Abuna Gindeberet district to the east and by Amhara regional state to the north. There are 31 rural and urban ‘kebeles’ in the district. Total population of the district is estimated to be 147256, of which 128696 were rural and 18560 were urban. The reproductive age group women (15–49 years) were 32588. Total pregnant women were 5110 [35]. Before study area selection health managers and other non-governmental stake holder recommendation were assessed. Depending on the health planners, non-governmental organization and expert recommendation study area was selected. The study was conducted from October 10/2020 – November 10/2020.

### Study Design and Population

A community-based cross-sectional study was conducted. All pregnant women residing in the district were source population and all pregnant women residing in randomly selected kebele were considered as study populations. All pregnant women who have lived for six months and above in the study ‘kebele’ were included in the study. Critically ill and pregnant women with both arm deformities were excluded from the study.

### Sample size determination and sampling technique

#### Sample size determination

The sample size was calculated using single population proportion formula with the following assumptions: The 44.9% prevalence was taken from a study done on under nutrition and associated factors among pregnant women in Gumay district, Jimma Zone, South West Ethiopia [22] with 95% confidence interval the, margin of error (d = 5%).

$$n = \frac{{{{\left( {z\alpha {/_2}} \right)}^2} \times p(1 - p)}}{{{d^2}}} = n = \frac{{{{\left( {z\alpha {/_2}} \right)}^2} \times pq}}{{{d^2}}} = n = \frac{{{{\left( {1.96} \right)}^2} \times 0.449 \times (1 - 0.449)}}{{{{\left( {0.05} \right)}^2}}} = 380$$

Where: n = required sample size.

Z = z -value corresponding to a 95% level of significance = 1.96.

d = margin of error (5%).

By considering 1.5 design effect and 10% non-response rate the final sample size for study were 627.

#### Sampling technique and sampling procedures

**Fig. 1:**
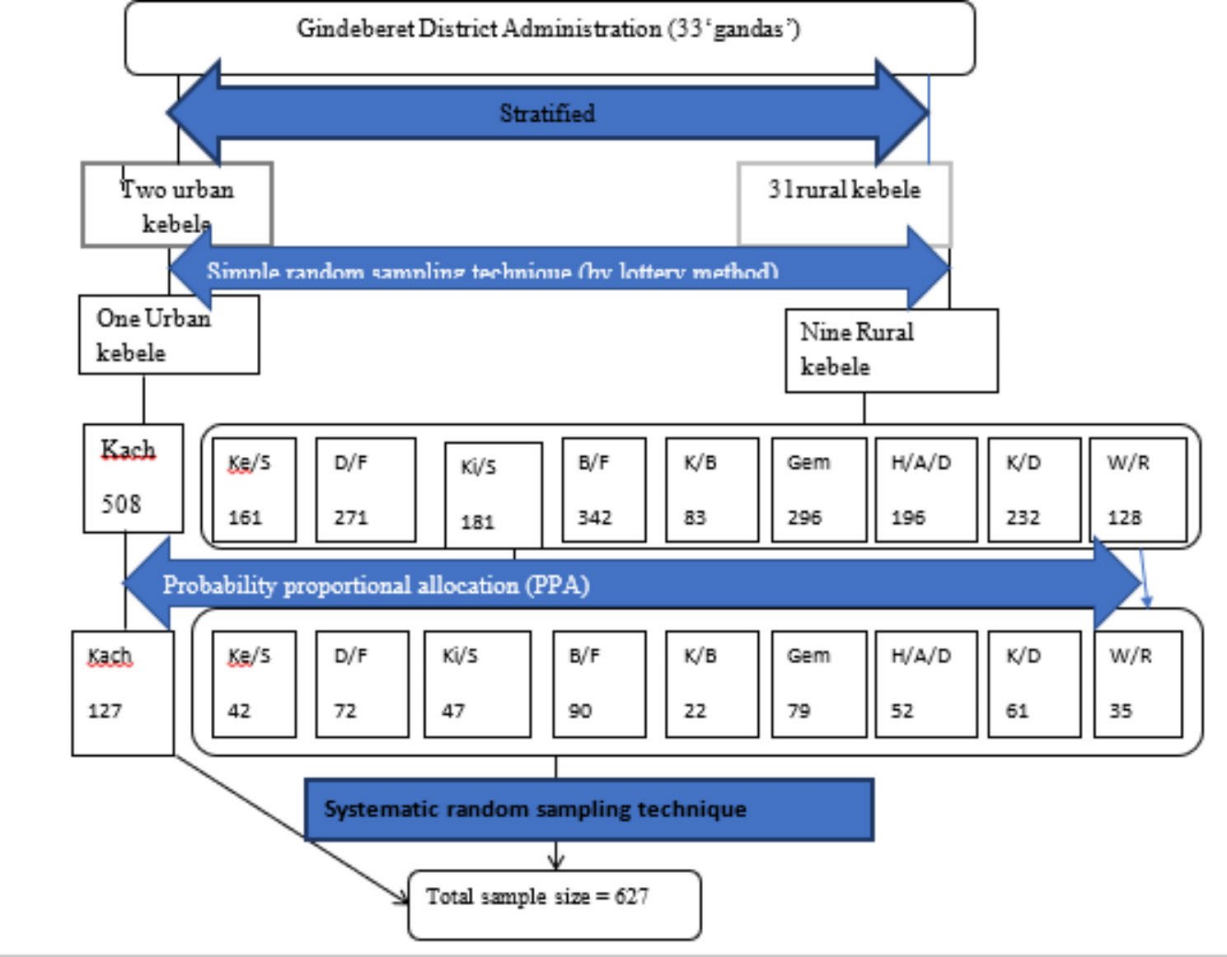
Schematic presentation of sampling procedure for the study on dietary diversity, under nutrition and associated factors among pregnant women in Gindeberet district, Oromia, Ethiopia, 2020 Key: PW = pregnant women, Kach = Kachise, Ke/S = Kere Sole, D/F = Dire Faji, Ki/S = Kiltu Senbeta, B/F = Beke Feyina, K/B = Kelo Bedasa, H/A/D = Hula Aba Dadi, K/D = Kere Dobi, W/R = Wine Roge

First, ‘kebeles (small administrative unit in Ethiopia)’ in the district were stratified into urban and rural areas. Then, 9 (nine) ‘kebeles’ from 31 rural and 1(one) ‘kebeles’ from two urban ‘kebeles’ were selected by lottery methods. Then, pregnant women were selected from each ‘kebele’ by systematic random sampling techniques using pregnant women registration book found in health post by considering a list of them as a sampling frame. Then, k was calculated as follows (K = N/n = 2400/627 = 3.8 ≈ 4; Where N = pregnant women in the selected kebele and n = total sample size required). Then, from 1 to 4 random start were selected by lottery methods. The random start 2 were selected, then every 4th pregnant women were selected from pregnant women registers until fulfilling the required sample size Fig. 1.

### Operational and term definitions

Undernutrition: MUAC less than 23 cm was an indicator of undernutrition and MUAC ≥ 23 cm was for normal nutritional status [36, 37].

Pregnant women: The women identified as pregnant by health extension workers and registered on pregnant women registration book.

Drinking water sources: ‘Protected’ if pipe/hand pump/protected spring and not otherwise [22].

Dietary diversity Score (DDS): sum of food groups eaten by pregnant women over 24 h preceding data collection [38].

Inadequate dietary diversity: when pregnant women consume less than five food groups.

Adequate dietary diversity: When pregnant women consume five or more food groups [39].

### Data collection tool and procedure

Data were collected by using pre-tested structured interviewer administered questionnaires through face-to-face interview and MUAC measuring tape. The questionnaires contain socio-demographic factors, obstetric characteristics of pregnant women, pregnant women dietary diversity and environmental factors. The questionnaires were adapted by reviewing different related literatures [36, 37, 40, 41].

The pregnant women dietary diversity was measured by a qualitative recall of all foods consumed by each pregnant women during the previous 24 h, which were validated tools prepared by FAO. It is a dichotomous indicator of whether or not to feed ≥ 5 of 10 food groups in the last 24 h. This was categorized as inadequate dietary diversity score (< 5 food groups) and adequate dietary diversity score (≥ 5 food groups). The ten lists of food groups were used to assess the 24 h recall [starchy staples, pulses, nuts/seeds, dairy, meat/poultry/fishes, eggs, dark green leafy vegetables, other vitamin-A rich fruits/vegetables, other fruits and other vegetables] [38].

Anthropometric measurements: trained data collectors measured a Mid Upper Arm Circumference (MUAC) according to standard. The MUAC of pregnant woman was measured at mid-point between the tip of the shoulder (olecranon process) and tip of the elbow (acromion process) of left arm. An adult MUAC tape that was non-elastic and non-stretchable was used to take measurements, after checking that the tape was applied without any clothing and with correct tension (not too loose or not too tight) [36, 37]. Data were collected by ten diploma nurses.

### Data quality control

Data were collected by structured and pretested questionnaires. pre-test was done on 5% of sample size in Damota kebele of the district other than selected kebele. The data collectors and supervisors were trained on how to approach pregnant women and collect data for two days. Language experts translate questionnaires to local language Afan Oromo and back to English to check consistency. MUAC was measured two times if there is variation between measurements average was accepted.

### Data Processing and Analysis

After data collection all questionnaires were checked for consistence and completeness before undergoing further analysis. Then data were coded and entered into Epi-info version 7.2.2.6 and exported to Statistical Package for Social Science (SPSS) version 23 for further data analysis. Descriptive statistics such as frequency, proportion, mean and standard deviation were used to describe characteristics of study participants. The presence of multicollinearity between independent variables was checked by using the variance inflation factor (VIF). However, there was no multicollinearity problems. Bi-variable and multivariable logistic regression analysis were carried out to identify factor associated with undernutrition and inadequate dietary diversity. The model fitness for the variables was assessed by the Hosmer-Lemeshow goodness of fit test statistics at p > 0.05, which shows fitness of the model. Odds ratios with their 95% confidence intervals were used to see strength of association between independent and dependent variables. Variables with P < 0.05 were considered as significantly associated with undernutrition and dietary diversity score.

## Results

### Socio-demographic factors

Six-hundred twenty-one pregnant women were included in the study with response rate of 99%. The mean age of pregnant women were 30 (± 5.4) years, while age of participants ranges from 18 to 45 years. Considering educational and occupational status of women, 163 (26.2%) of participants were able to read and write and 505 (81.3%) of participants were housewife. 494 (79.5%) of the pregnant women were rural resident. 336(54.1%) and 285 (45.9%) of study participant had family size of ≥ 5 and < 5 children respectively Table 1.

**Table 1:** Socio-demographic factors among pregnant women in Gindeberet district, Oromia, Ethiopia, 2020 (n = 621)

Variables	Categories	Frequency	Percent (%)
Age of participants	< 20	113	18.2
	20–35	399	64.3
	> 35	109	17.6
Place of Residence	Urban	127	20.5
	Rural	494	79.5
Religion	Orthodox	154	24.8
	Protestant	459	73.9
	Wakefeta	8	1.3
Ethnicity	Oromo	616	99.2
	Other	5	0.8
Marital status	Married	540	87.0
	Others**	81	13.0
Occupational status of the mother	Farmer	18	2.9
	House wife	453	72.9
	Private employee	51	8.2
	Merchant	43	6.9
	Government employee	56	9.0
Occupational status of the husband	Farmer	422	68.0
	Private employee	49	7.8
	Merchant	63	10.1
	Government employee	87	14.0
Educational status of mother	Informal education	213	34.3
	Only primary education (1–8 grade)	224	36.1
	Secondary education (9–12 grade)	132	21.3
	College Diploma and above	52	8.4
Educational status of husband	Informal education	187	30.1
	Only primary education (1–8 grade)	134	21.6
	Secondary education (9–12 grade)	194	31.2
	College Diploma and above	106	17.1
Number of family size	< 5	285	45.9
	≥ 5	336	54.1
Number of family size < 15 years	Number of family who have no < 15 years children	67	10.8
	Number of family who have < 15 years children	554	89.2
Number of family size > 65 years	Number of family who have no > 65 years	529	85.2
	Number of family who have > 65 years	92	14.8

** Amhara, Tigre

### Obstetrics related factors

About 153 (24.6%) of participants had their first pregnancy at teenage (< 20 years). With regard to gravidity 515 (82.9%) of study participants had less than five pregnancies before current and 66 (10.6%) of study participants had never been pregnant before current pregnancy. 322 (51.9%) and 299 (48.1%) of study participants had birth interval of greater and less than three years respectively. With regard to trimester of pregnancy, 373 (60.1%) and 186 (30.0%) of study participants were in second and third trimester of pregnancy respectively. Majority of the pregnant women had first ANC visit and second ANC visit which were 146(23.5%) and 242(39%) respectively. About 45(7.2%) of the women had history of one abortion Table 2.

**Table 2:** Obstetrics characteristics among pregnant women in Gindeberet district, Oromia, Ethiopia, 2020 (n = 621)

Variables	Categories	Frequency	Percent (%)
Gestational week	First trimester (1–12) weeks	62	10.0
	s trimester (13–27) weeks	373	60.1
	Third trimester (28–40) weeks	186	30.0
Ever been pregnant before	No	66	10.6
	Yes	555	89.4
Gravidity	< 5	515	82.9
	≥ 5	106	17.1
Parity	0	75	12.1
	1–3	228	36.7
	≥ 4	318	51.2
Year of inter-pregnancy interval	< 3	299	48.1
	≥ 3	322	51.9
Attending ANC clinic	No	97	15.6
	Yes	524	84.4
ANC Visit	First visit	146	23.5
	s visit	242	39.0
	Third visit	103	16.6
	Fourth and above visit	31	5.9
Using of modern contraceptive before	No	148	23.8
	Yes	473	76.2
Age at first pregnancy	< 20 years	153	24.6
	21–30 years	464	74.7
	> 30 years	4	0.6
Numbers of abortions made	0	572	92.1
	1	45	7.2
	≥ 2	4	0.6
Intention of pregnancy	Planned	532	85.7
	Unplanned	89	14.3

### Dietary consumption related factors

Majority of respondents 503 (81%) and 118 (19%) ate meals less than four and greater or equal to four times a day respectively. More than half of respondents 377 (60.7%) took two cups of coffee per day. Almost all of respondents 559 (90.0%) did not consume alcohol during their pregnancy. 440(70.9%) of pregnant women did not change their feeding style during pregnancy and 153(24.6%) had eating problem during pregnancy. Around 181(29.1%) of study participant had nutritional information Table 3.

**Table 3:** Dietary consumption related factors among pregnant women in Gindeberet district, Oromia, Ethiopia, 2020 (n = 621)

Variables	Categories	Frequency	Percent (%)
Alcohol consumption	Yes	62	10
	No	559	90.0
Frequency of Coffee consumption	None (Don’t take coffee at all)	44	7.1
	Rarely (once a week or less)	12	1.9
	Seldom (once in a day)	188	30.3
	Often ( > = two cups per day)	377	60.7
Changing feeding style after	No	440	70.9
	Yes	181	29.1
Frequency of meals	Less than four times per day	503	81
	Four and above times per day	118	19
Having Nutritional information	No	181	29.1
	Yes	440	70.9
Sources of nutritional information	Health provider	365	58.8
	Family	2	0.3
	Media	69	11.1
	Friends	3	0.5
Having eating problem	No	468	75.4
	Yes	153	24.6
Type of eating problem	Loss of appetite	54	8.7
	Vomiting	57	9.2
	Nausea	57	9.2
	Heart burn	23	3.7

### Environmental related factors

556 (89.5%) of respondents had latrine. The majority of respondents 570 (91.8%) used drinking water from protected sources and about 355 (57.2%) of respondents washed their hands after latrine. Regarding hand washing during critical time 97.6%, 68% and 57.2% of women washed their hand before eating, before food preparation and after toilet respectively Table 4.

**Table 4:** Environmental related factors among pregnant women in Gindeberet district, Oromia, Ethiopia, 2020 (n = 621)

Variables	Categories	Frequency	Percent (%)	
Source of drinking water	Protected sources	570	91.8	
	unprotected sources	51	8.2	
Latrine possession	No	65	10.5	
	Yes	556	89.5	
Critical times of hand washing practice	After latrine	355	57.2	
	Before food preparation	422	68.0	
	Before eating	606	97.6	
	After clean child feces	383	61.7	
	After handling garbage	612	98.6	

### Prevalence of undernutrition

**Fig. 2:**
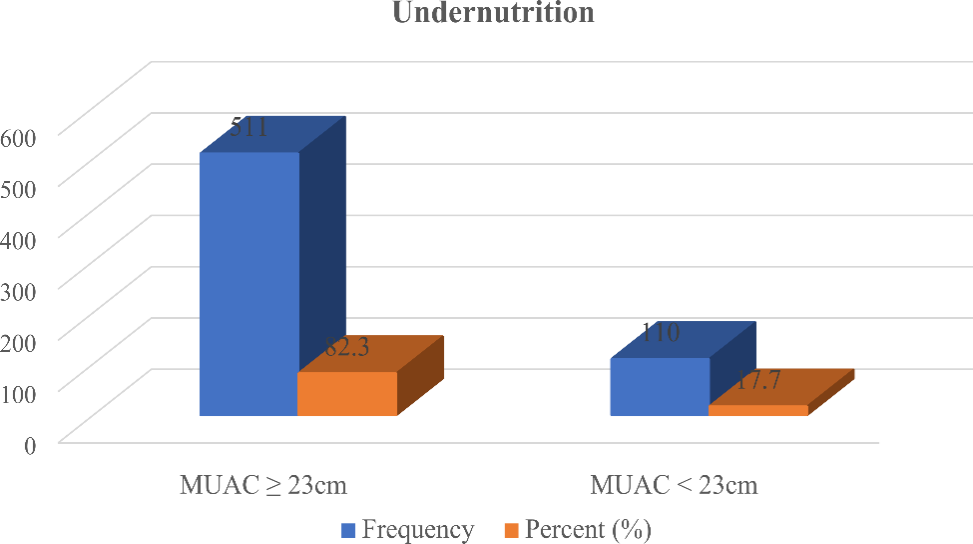
Prevalence of undernutrition among pregnant women in Gindeberet district, Oromia, Ethiopia, 2020 (n = 621)

Over all prevalence of undernutrition were 110 (17.7%) among pregnant women in study area. The mean of MUAC measurement among pregnant women was 23.99 cm (± 1.62 SD) Fig. 2.

### Prevalence of Dietary Diversity

**Fig. 3:**
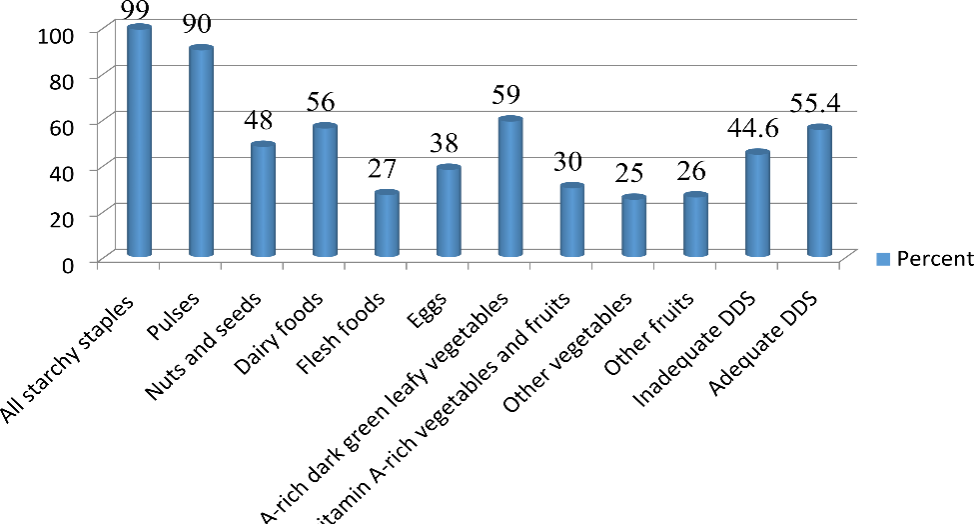
Dietary diversity among pregnant women in Gindeberet district, Oromia, Ethiopia, 2020 (n = 621)

The mean dietary diversity score of study participant were 4.98 (± 1.26SD). Among the participant 345 (55.4%) and 276 (44.6%) had adequate and inadequate dietary diversity score respectively. Regarding the consumed food groups by pregnant women in the previous 24 h, nearly all women 615 (99%) consumed starchy staples, 560 (90.2%) of women consumed pulses, and 325 (52.3%) consumed nuts and seeds. Moreover, 169 (27.2%) flesh foods were minimally consumed and other vegetables 154 (24.8%) were the least consumed food groups Fig. 3.

### Factors associated with undernutrition

Bivariable and multivariable logistic regression analysis were carried out to identify factor associated with undernutrition. On multivariable analysis ANC visit, family size and source of drinking water were significantly associated with undernutrition.

This study showed that pregnant mothers who did not received antenatal care (ANC) during their pregnancy were two times more likely [AOR = 2.32, 95% CI: (1.38, 3.90)] to be undernourished when compared to their counterpart. Pregnant women who had ≥ 5 family size [AOR: 2.93; 95% CI: (1.10, 7.79)] were three times more likely to develop undernutrition when compared to study participant who had < 5 family size. The pregnant women used unprotected sources of water [AOR: 4.14, 95% CI: (1.63, 10.52)] were four times more likely to develop undernutrition when compared to those who use protected source of water Table 5.

**Table 5:** Bi-variable and multivariable logistic regression analysis of factors associated with undernutrition among pregnant women in Gindeberet district, Oromia, Ethiopia, 2020 (n = 621)

Variables		Undernutrition		COR (95% CI)	AOR (95% CI)	P-value
		Yes	No			
Education of mothers	Informal education	41(20%)	156(80%)	0.25(0.06, 0.95)	0.526(0.07, 3.85)	0.527
	Primary school	36(16.1%)	188(83.9%)	0.32(0.09, 1.08)	0.49(0.08, 3.21)	0.458
	Secondary school	22(16.7%)	110(83.3%)	0.31(0.087, 1.07)	0.59(0.94, 3.68)	0.570
	Diploma & above	11(16.18%)	57(83.82%)	1	1	
Occupation of mothers	Farmer	11(50%)	11(50%)	4.60(1.56, 13.54)	1.45(0.11, 20.06)	0.781
	Housewife	67(14.9%)	382(85.1%)	0.81(0.39, 1.68)	2.21(0.26, 18.56)	0.465
	Private employee	16(30.8%)	35(69.2%)	2.04(0.69, 6.00)	11.80(1.20, 53.84)	0.134
	Merchant	6(14%)	37(86%)	0.75(0.25, 0.24)	1.63(0.17, 15.86)	0.676
	Gov’t employee	10(17.9%)	46(82.1%)	1	1	
Residence	Urban	11(8.7%)	116 (91.3%)	1	1	
	Rural	99(20%)	395(80%)	2.64(1.371, 5.09)	1.30(0.421, 4.02)	0.648
Source of drinking water	Protected	95(16.7%)	475(83.3%)	1	1	
	Unprotected	15(29.4%)	36(70.6%)	2.08(1.10, 3.96)	4.14(1.63, 10.52)	0.003*
ANC visit	Yes	444(84.7%)	80(15.3%)	1	1	
	No	67(69.1%)	30(30.9%)	2.49(1.519, 4.07)	2.32(1.38, 3.90)	0.001*
Gravidity	≤ 5	45(15.5%)	245(84.5%)	1		
	> 5	65(19.6%)	266(80.4%)	0.75(0.49, 1.14)	0.73(0.38, 1.43)	0.367
Gestational age	First trimester	15(24.2%)	47(75.8%)	1	1	
	s trimester	65(17.4%)	308(82.6%)	0.66(0.35, 1.25)	0.633(0.32, 1.26)	0.193
	Third trimester	30(16.1%)	156(83.9%)	0.60(0.30, 1.21)	0.67(0.32, 1.43)	0.305
Having nutrition information	Yes	66(15.0%)	374(85.0%)	1	1	
	No	44(24.3%)	137(75.7%)	1.82(1.19, 2.79)	1.280(0.77, 2.13)	0.341
Family size	> 5	69(20.5%)	267(79.5%)	1.54(1.10, 2.35)	2.93(1.10, 7.79)	0.031*
	≤ 5	41(14.5%)	244 (85.5%)	1		
Alcohol consumption	Yes No	57(91.9%) 454(81.2%)	5(8.1%) 105(18.8%)	0.38(0.15, 0.97) 1	2.74(1.05, 7.17) 1	0.054

*Significant at p-value < 0.05, COR = Crude Odds Ratio, AOR = Adjusted odds ratio, C.I.=Confidence Interval

### Factors Associated with inadequate Dietary Diversity

Multivariable logistic regression analysis showed that place of residence, ANC visit and nutrition information were significantly associated with dietary diversity score. This study revealed that pregnant women who live in rural area were [AOR = 2.59, 95% CI: (1.66, 4.04)] 2.59 times more likely to have inadequate dietary diversity than those who live in urban area. Pregnant women who did not visit ANC [AOR = 2.52, 95% CI: (1.58, 4.03)] were 2.52 times more likely to have inadequate dietary diversity than those who visit ANC during their pregnancy. Pregnant women who had nutrition information [AOR 1.43; 95% CI: (1.10, 2.10)] were 1.43 times more likely to have inadequate dietary diversity, as compared to those who did not have nutrition information Table 6.

**Table 6:** Bi-variable and multivariable logistic regression analysis showing factors associated with dietary diversity among pregnant women in Gindeberet district, Oromia, Ethiopia, 2020 (n = 621)

Variables		Dietary diversity		COR (95% CI)	AOR (95% CI)	P-value
		Adequate	Inadequate			
Education of mothers	Informal education	115(56%)	98(44%)	3.30(1.36, 8.02)	0.56 (0.12, 2.55)	0.452
	Primary school	116(51.8%)	108(48.2%)	3.91(1.87, 8.18)	0.65 (0.16, 2.65)	0.543
	Secondary school	72(54.5%)	60(45.5%)	3.50(1.62, 7.56)	0.70 (0.18, 2.73)	0.604
	Diploma & above	42(80.8%)	10(19.2%)	**1**	**1**	
Occupation of mothers	Farmer	9(40.9%)	13(59.1%)	3.61(1.29, 10.10)	2.87(0.75, 11.01)	0.125
	Housewife	244(54.3%)	205(45.7%)	2.10(1.14,3.86)	2.28(0.89, 5.83)	0.085
	Private employee	29(61.5%)	22(38.5%)	1.56(0.59, 4.16)	0.79(0.13, 4.82)	0.804
	Merchant	23(53.5%)	20(46.5%)	2.17(0.94, 5.00)	2.55(0.88, 7.40)	0.085
	Gov’t employee	40(71.4%)	16(28.6%)	**1**	**1**	
Place of residence	Urban	94(74%)	33(26%)	**1**	**1**	
	Rural	251(50.8%)	243(49.2%)	2.76(1.79, 4.26)	2.59(1.66, 4.04)	0.001*
Source of drinking water	Protected sources	311(54.6%)	259(45.4%)	**1**	**1**	
	Unprotected sources	34(66.7%)	17(33.3%)	0.60(0.33, 1.10)	0.44(0.24, 0.83)	0.125
ANC visit	Yes	310(59.2%)	214(40.8%)	1	**1**	
	No	35(36.1%)	(6263.9%)	2.57(1.64,4.02)	2.52(1.58, 4.03)	0.001*
Having nutrition information	Yes	267(60.7%)	173(39.3%)	1	**1**	
	No	78(43.1%)	103(56.9%)	2.04(1.44, 2.89)	1.43(1.10, 2.10)	0.001*
Consumption of alcohol	Yes	46(74.2%)	16(25.8%)	2.50(1.38, 4.52)	2.32(1.24, 4.33)	0.065
	No	299(53.5%)	260(46.5%)	1		
Vomiting	1. Yes	27(47.4%)	30(52.6%)	0.70(0.40, 1.20)	0.76(0.42, 1.38)	0.363
	2. No	318(56.4%)	246(43.6%)	1	1	
Gravidity	1. < 5 2. ≥ 5	288(55.9%) 57(53.8%)	227(44.1%) 49(46.2%)	1	1 0.84(0.53, 0.31)	0.44

*Significant at p-value < 0.05, COR = crude odds ratio, AOR = Adjusted odds ratio, C.I = Confidence Interval

## Discussion

This study assessed prevalence of undernutrition, dietary diversity and associated factors among pregnant women in Gindeberet district, Oromia, Ethiopia. In the current study prevalence of undernutrition and inadequate dietary diversity were 110 (17.7%) and 276 (44.6%) respectively. ANC visit, family size and source of drinking water were significantly associated with undernutrition, where as place of residence, ANC visit and nutrition information were significantly associated with inadequate dietary diversity score.

The study found that 110 (17.7%) of pregnant women were undernourished. This is in line with study done in Dire Dawa city administration, Ethiopia (18.2%), Gondar Town Northern Ethiopia (14.4%) and Laikipia, Kenya (19.3%) [16, 23, 39]. Our finding was lower than the study conducted in Syrian, Jordan (49.2%) [42]. The difference could be related to difference in socio-demographic characteristics of study participants, it is also lower than the finding from Addis Ababa (24.6%), Shashemene district west Arsi zone (34%), Gumay district Jimma zone (44.4%) [20, 22, 43]. These differences may be due to variations in supply and access of dietary diversity and nutrition information, sample size difference and study time zone variation.

Our study found 276 (44.6%) of pregnant women had inadequate dietary diversity. This is higher than the study done in Laikipia, Kenya (39.2%), Ghana (14.5%), Tigray, Ethiopia (38.8%), North East Ethiopia (31.4%), Shashamane, Ethiopia (25.4%) [23, 25, 44–46]. The possible difference might be related to difference in time zone variation of study and sample size difference. Furthermore, this study contains 10 food groups with two categories whereas study conducted in Ghana contained eleven food groups. Additionally, geographical location, seasonal variability and socio-cultural factors may result in difference.

Our finding on inadequate dietary diversity was lower than study done in Gojjam, Northwest Ethiopia (55%), Dire Dawa town, Ethiopia (57%), Bale, Oromia, Ethiopia (54.5%) [17, 26, 27]. The difference might be due to study period, variation of the food group involved in assessing dietary diversity, as study conducted in Bale, Oromia, Ethiopia contained nine food groups. Also, variation in geographical location and agricultural practice may be making the difference.

The study showed that, pregnant women who did not visit antenatal care (ANC) during their pregnancy were 2 times more likely to be undernourished when compared to mothers who visit ANC. This finding was in line with study conducted in Shashemene district west Arsi zone and Rayitu district Bale zone of Oromia, Ethiopia [20, 26]. This might be due to the fact that pregnant women who visit ANC clinic have more information on proper consumption of diet during their pregnancy.

Pregnant women who had family size greater than five were more likely to be undernourished than pregnant women who had family size equal or less than five. This finding was supported by a study conducted in Madagascar and Ilu Aba Bor zone, southwest Ethiopia [29, 47]. This might be due to the fact that women with large family sizes share meals (foods) with other family members, thus, pregnant women might not get enough foods. As a result, they prone to develop undernutrition.

Our finding also showed that pregnant women those used unprotected sources of water were 4 times more likely to be undernourished than those mothers who used protected sources of water. The possible reason could be explained as pregnant women use non-potable water, they became susceptible to infectious diseases which may disturb digestive system and subsequently leads to malabsorption. This leads to the fact that women with malabsorption may develop undernutrition.

Pregnant women who lived in rural were two times more likely to have inadequate dietary diversity when compared to their counterparts. This finding was consistent with study conducted in Fitche, Oromia, Ethiopia [48] this could be related to lifestyle difference between rural and urban resident and easy accessibility of different food groups by urban residents.

Pregnant women who had not nutrition information during their pregnancy were 1.43 times more likely to have inadequate dietary diversity when compared to those who had nutrition information. This is also supported by study conducted in North East Ethiopia, west Shoa, Oromia, Ethiopia and Addis Ababa, Ethiopia [2, 49, 50]. This may be due to the fact that those who get information about nutrition had better knowledge and understanding to practice diversified diet than those who did not have nutrition information.

Pregnant women who did not visit ANC clinic were two times more likely to have inadequate dietary diversity when compared to those who visited the clinic. This could be due to the fact that ANC visits offer a contact point to get nutritional counsel for pregnant women as well as during ANC visit women may get information on different locally available food source as well as advised on importance of diversified meal during pregnancy.

## Conclusions

Magnitude of undernutrition and inadequate dietary diversity among pregnant women were high in study area. Source of drinking water, ANC visit and family size were significantly associated with pregnant women undernutrition and place of residence, ANC visit and nutrition information were significantly associated with inadequate dietary diversity. Therefore, pregnant women, government, non-governmental organization and other stake holders should focus on importance of ANC visit and adequate dietary diversity to improve nutritional status of pregnant women.

### Recommendations

Based on the finding the following recommendation was forwarded.

#### For woreda health office and health extension workers

- To give health education on importance of ANC visit to improve nutritional status of women.
- To provide counselling on importance of child spacing to improve nutritional status of pregnant women.
- To educate pregnant women on diversified meal, early initiation of ANC and clean source of water during pregnant women conference.

#### For pregnant women

- To initiate Antenatal care early.
- To use clean source of water.
- To practice diversified meal from locally available source.

## Data Availability

The data and all supporting materials used in the preparation of this manuscript are freely available from the corresponding author at reasonable request.

## Abbreviations

CI: Confidence Interval
DDS: Dietary Diversity Score
EDHS: Ethiopian Demographic and Health Survey
FAO: Food and Agriculture Organization
IUGR: Intra Uterine Growth Restriction
MUAC: Mid-Upper-Arm-Circumstance
SD: Standard Deviation
SGA: Small for Gestation Age
SPSS: Statistical Package for Social Science
WHO: World Health Organization
